# Psychobehavioral Responses and Likelihood of Receiving COVID-19 Vaccines during the Pandemic, Hong Kong

**DOI:** 10.3201/eid2707.210054

**Published:** 2021-07

**Authors:** Kin On Kwok, Kin Kit Li, Arthur Tang, Margaret Ting Fong Tsoi, Emily Ying Yang Chan, Julian Wei Tze Tang, Angel Wong, Wan In Wei, Samuel Yeung Shan Wong

**Affiliations:** The Chinese University of Hong Kong, Hong Kong (K.O. Kwok, M.T.F. Tsoi, E.Y.Y. Chan, A. Wong, W.I. Wei, S.Y.S. Wong);; City University of Hong Kong, Hong Kong (K.K. Li);; Sungkyunkwan University, Seoul, South Korea (A. Tang);; University of Leicester, Leicester, UK (J.W.T. Tang)

**Keywords:** SARS-CoV-2, COVID-19, coronavirus, 2019 novel coronavirus disease, severe acute respiratory syndrome coronavirus 2, zoonoses, coronavirus disease, viruses, psychobehavioral, risk perception, preventive measures, vaccine uptake, longitudinal assessment, vaccines, Hong Kong

## Abstract

To access temporal changes in psychobehavioral responses to the coronavirus disease (COVID-19) pandemic, we conducted a 5-round (R1–R5) longitudinal population-based online survey in Hong Kong during January–September 2020. Most respondents reported wearing masks (R1 99.0% to R5 99.8%) and performing hand hygiene (R1 95.8% to R5 97.7%). Perceived COVID-19 severity decreased significantly, from 97.4% (R1) to 77.2% (R5), but perceived self-susceptibility remained high (87.2%–92.8%). Female sex and anxiety were associated with greater adoption of social distancing. Intention to receive COVID-19 vaccines decreased significantly (R4 48.7% to R5 37.6%). Greater anxiety, confidence in vaccine, and collective responsibility and weaker complacency were associated with higher tendency to receive COVID-19 vaccines. Although its generalizability should be assumed with caution, this study helps to formulate health communication strategies and foretells the initial low uptake rate of COVID-19 vaccines, suggesting that social distancing should be maintained in the medium term.

Since the World Health Organization declared coronavirus disease (COVID-19) a pandemic on March 11, 2020 (*1*), COVID-19 has infiltrated every continent in the world (*2*). Hong Kong, a densely populated city located on the southern coast of China with ≈7.5 million citizens and a mean daily number of 12.5 social encounters per individual (*3*), recorded its first laboratory-confirmed COVID-19 case in late January 2020 (*4*). Since then, Hong Kong has been adopting a suppress-and-lift strategy, under which lifting and reimposing of restrictions occurred based on epidemiologic thresholds (*5*). As of April 9, 2021, Hong Kong had recorded 11,550 confirmed cases and 205 deaths (crude case-fatality rate 1.8%) (*6*), and the fourth wave of COVID-19 epidemic had just ended. After more available data on phase 3 clinical trials of candidate vaccines (*7*) became available and the vaccine was authorized for emergency use, the COVID-19 vaccination program in Hong Kong began in late February 2021.

Surveillance of psychobehavioral responses during the epidemic plays an essential role to convey risk communication messages to the public. Previously, we reported that the general population in Hong Kong had high levels of perceived risk and mild anxiety during the early phase of the COVID-19 epidemic; the prompt government interventions with widely adopted individual precautionary measures might be the determinants to slow down the transmission early in the outbreak (*8*). After that initial analysis, which was based on cross-sectional data (*8*), global researchers have applied similar protocols for the general public in Japan (*9*), Saudi Arabia (*10*), Italy (*11*) and the United Kingdom (*12*). However, the temporal variations of psychobehavioral responses have not been examined.

In addition to psychobehavioral responses, unique to COVID-19 is its unprecedented massive epidemic size compared with other recent outbreaks, such that vaccination becomes the exit strategy. However, despite vaccine availability, vaccine doubters may hamper the global effort against COVID-19 (*13*). Unraveling the reasons behind vaccine hesitancy and monitoring its trends over time will support the design of interventions to boost COVID-19 vaccine uptake.

We report a longitudinal analysis of 5 representative population-based surveys of adults in Hong Kong on their psychological, behavioral, and vaccine-related responses, conducted during the first 2 waves of the COVID-19 epidemic. Our main objectives were tracking major psychobehavioral responses (including risk perception, psychological distress, and adoption of precautionary measures) over time and examining the determinants of the intention to receive the COVID-19 vaccine. As a complement, other psychobehavioral responses (such as knowledge about COVID-19) were also reported. These findings should have major implications for infection control policies and targeted mental health recommendations. Hong Kong has a high-income economy but had major social unrest in the prepandemic period in the population (*14*); thus, the experience in Hong Kong may act as a reference for other similar populations to prepare for future epidemics.

## Methods

### Respondent Recruitment

We established a community cohort within 36 hours after the first COVID-19 confirmed case was identified in Hong Kong. District councilors shared an online survey link through channels in which they convey information to their targeted residents (*8*). We arranged 5 follow-up rounds (denoted as R1–R5) of online surveys of the community cohort during January–September 2020, each lasting for 3–6 weeks: R1, January 23–February 13; R2, March 6–April 14; R3, May 8–June 14; R4, July 15–August 7; and R5, August 8–September 15. Respondents were compensated with cash vouchers in Hong Kong dollars (HKD): HKD 10 for R1, HKD 20 for R2), and HKD 30 for R3–R5).

### Study Instrument

The study instrument was based on a questionnaire used during the initial phase of the COVID-19 epidemic in Hong Kong (*8*). In each round, we administered questions soliciting key information on demographics, health conditions, travel history, risk perceptions toward COVID-19, anxiety and burnout, confidence in the local government and doctors, knowledge about COVID-19 transmission, and adoption and perceived effectiveness of preventive measures. In response to the funding commitments for COVID-19 vaccine development (*15*), starting with R4, we embedded vaccine-related questions along 2 dimensions: intention to receive COVID-19 vaccines when available and vaccine hesitancy. 

#### Psychological Responses

Risk perceptions toward COVID-19 included perceived susceptibility (of oneself and one’s family members), assuming no precautionary measure, and perceived severity. Starting with R3, we asked respondents to report their perceived susceptibility based on the situation during which they completed the survey (1, very likely; 5, very unlikely). In addition, respondents rated the level of disease severity of COVID-19 and other noncommunicable diseases and infectious diseases (1, very bad; 5, not bad at all).

We measured anxiety with the Chinese–Cantonese version of the Hospital, Anxiety and Depression Scale—Anxiety (*16*). Respondents rated 7 statements on the basis of their feelings in the preceding 7 days on a 4-point scale; a higher score indicated stronger anxiety (summative score: 0–7, normal; 8–10, borderline abnormal; 11–21, abnormal).

We measured burnout with a single-item measure: “Overall, based on your definition of burnout, how would you rate your level of burnout when facing COVID-19?” (1, “I have no symptoms of burnout”; 5, “I feel completely burned out and often wonder if I can go on facing COVID-19; I am at the point where I may need some changes or may need to seek some sort of help”). This single-item measure was refined from a nonproprietary validated burnout measure (*17*) to fit the current context and was asked starting with R3.

#### Behavioral Responses

Respondents rated the adoption (yes/no) (Appendix Table 1) and perceived effectiveness (1, very effective; 5, not very effective) (Appendix Table 2) of 17 precautionary measures against COVID-19. For the likelihood of COVID-19 vaccine uptake, respondents answered this question “If COVID-19 vaccines are available, how likely will you receive them?” (0, definitely not; 10, definitely). We measured vaccine hesitancy with a validated 15-item tool (*18*) with 3 items on each of 5 psychological antecedents (the 5Cs): confidence, defined as trust in the safety and effectiveness of the vaccine, the system that delivers the vaccine, and the motivations of policymakers who decide on the need of the vaccine; complacency, defined as not perceiving the disease as high risk and vaccination as necessary; constraints, defined as barriers to vaccination; calculation, defined as persons’ engagement in extensive information searching; and collective responsibility, defined as willingness to protect others through herd immunity. We used an average score for each antecedent. For collective responsibility, one reverse item, “When everyone is vaccinated, I don’t have to get vaccinated, too,” was excluded from the calculation. The vaccine-related items did not include any specific information about pharmaceutical companies or manufacturing countries.

### Statistical Analysis

We summarized responses using descriptive statistics. To examine the overall linear trends in the responses and to account for the correlation diminishment resulting from responses from the same respondents across time, we adopted the generalized estimation equation framework featuring an autoregressive structure for within-subject correlations. To compare the proportion of responses in 2 time points, we used a partially overlapping samples *z*-test (*19*).

We adopted a multivariate regression model under the generalized estimation equation framework to identify the associated factors for higher tendency for social distancing (i.e., >5 social distancing measures) and higher uptake tendency for COVID-19 vaccines (i.e., >7 of 10 for the “likelihood of receiving COVID-19 vaccines” question). We reported adjusted odds ratios (aORs) and 95% CIs and specified a statistical significance of p<0.05. We conducted the analysis in R software version 4.0.3 (https://www.r-project.org). This study was approved by the Survey Behavioral Research Ethics Committee of The Chinese University of Hong Kong (reference no. SBRE-20-037).

## Results

### Study Timeline

**Figure 1:**
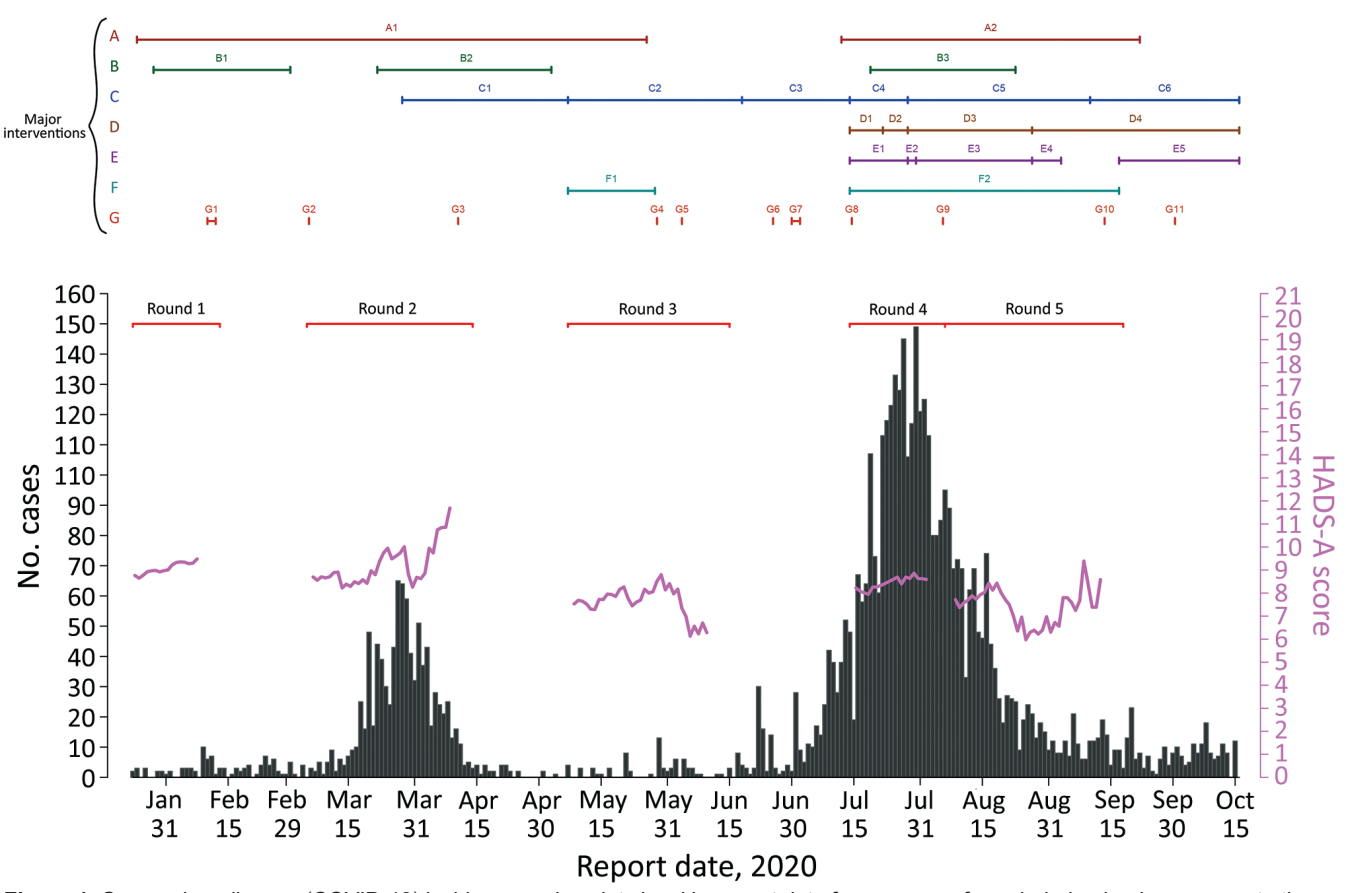
Coronavirus disease (COVID-19) incidence and anxiety level by report date from survey of psychobehavioral responses to the COVID-19 pandemic, showing timeline of major interventions, Hong Kong, 2020. A, school closures: A1, closure, Jan 25–May 26; A2, early start of summer holiday, Jul 13–Sep 22. B, government work-from-home arrangement: B1, Jan 29–Mar 1; B2, Mar 23–May 3; B3, Jul 20–Aug 23. C, group size limits on gatherings in public places: C1, limit 4, Mar 29–May 7; C2, limit 8s, May 8–Jun 18; C3, limit 50, Jun 19–Jul 14; C4, limit 4, Jul 15–Jul 28; C5, limit 2, Jul 29–Sep 10; C6, limit 4 persons, Sep 11–present. D, compulsory mask wearing: D1, on public transportation, Jul 15–present; D2, also in public indoor areas, Jul 23–present; D3, also in public outdoor areas, Jul 29–present (exemption for country parks or when engaging in strenuous physical activities in public outdoor spaces, Aug 28–present). E, regulations applied to restaurants, Mar 28–present: <50% of premises capacity; tables >1.5 m apart; no more than 2, 4, or 8 persons per table; compulsory mask wearing except when consuming food or drink; compulsory body temperature screening before entry; hand sanitizer on premises; suspension of dine-in service for the following periods: E1, 6 pm–4:59 am, Jul 15–Jul 28; E2, at all times, Jul 29–30; E3, 6 pm–4:59 am, Jul 31–Aug 27; E4, 9 pm–4:59 am, Aug 28–Sep 3; E5, 12 am–4:59 am, Sep 18–present. F, business closures: F1, bathhouses, party rooms, clubs, karaoke clubs, May 8–May 28; F2, bathhouses, party rooms, clubs, karaoke clubs (all reopened Sep 17), swimming pools (beginning Jul 29), sports premises (Jul 29–Aug 28), clubhouses (reopened Aug 28), beauty parlors (reopened Aug 28), massage establishments (reopened Sep 4), places of public entertainment (reopened Aug 28), places for amusement (reopened Sep 4), fitness centers (reopened Sep 4), and amusement game and mahjong-tin kau establishments (reopened Sep 11). G, vaccine development timeline: G1, World Health Organization (WHO) Convention of Global Research and Innovation, Feb 11–12; G2, WHO Global Research Roadmap prioritizing vaccine development, Jun 3; G3, draft landscape of candidate vaccines, Apr 11; G4, launch of COVID-19 Access Pool for sharing data for vaccine development, May 29; G5, funding commitment at Global Vaccine Summit for immunization in low-income countries, Jun 4; G6, call for USD 31.3 billion for therapeutics and vaccine development, Jun 26; G7, second summit on COVID-19 research and innovation, Jul 1–2; G8, engaging >150 countries in financing vaccines, Jul 15; G9, outline of global vaccine procurement, Aug 6; G10, WHO guidance on vaccine allocation between and within countries, Sep 14; G11, WHO calls for vaccine manufacturers to apply for prequalification, Oct 1. HADS-A, Hospital, Anxiety and Depression Scale—Anxiety.

**Figure 2:**
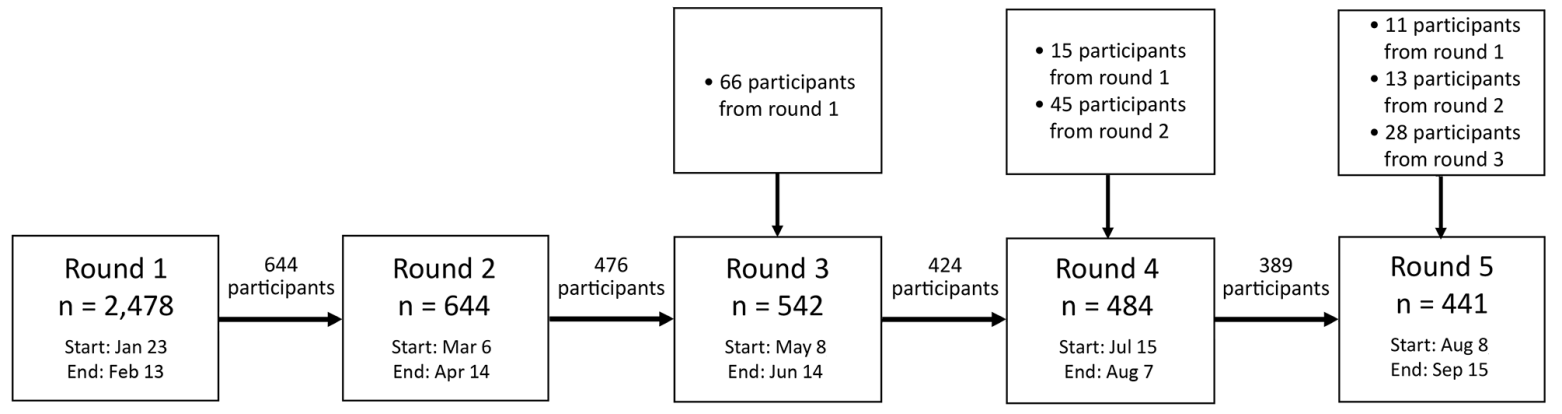
Timeline and participant recruitment for survey of psychobehavioral responses to the coronavirus disease pandemic, Hong Kong, 2020. To qualify for the survey, participants had to be >18 years of age and reside in Hong Kong for >5 days/week in the preceding month. The numbers in the box for each round refer to the number of respondents who indicated willingness to participate in the respective survey round; they may or may not have completed the questionnaire.

The 5 study rounds intertwined epidemic waves 1 and 2 in Hong Kong (*20*) at different disease stages (Figure 1): the initial phase (R1), amid epidemic waves (R2 and R4), during the relative quiescence between 2 waves (R3), and the decaying phase of wave 2 (R5). The government-initiated interventions (such as school closure and compulsory mask-wearing) and the call for COVID-19 vaccine were also presented (Figure 1). The data collection was completed before any announcement of the safety and efficacy trials of the candidate vaccines. We received 2,478 attempts to complete the survey in R1, of which 1,715 provided complete responses (*8*) and 1,054 indicated willingness to participate in future studies. The sample sizes for R2–R5 ranged from 441 to 644 (Figure 2).

### Respondent Characteristics

The cohort consisted of more female persons (68.5%–69.8%) and young adults (18–44 years of age) (78.6%–81.0%) than other categories. Most were well educated: 78.9%–82.5% had at least postsecondary level education (Appendix Table 3). Most respondents were free from chronic diseases (87.1%–88.8%) and perceived themselves to be in good health (73.1%–78.1%) (Appendix Table 4). 

### Risk Perception over Time

We identified significant temporal variation of risk perception toward COVID-19 (Appendix Table 5). Assuming no precaution measures, respondents perceived themselves likely to be infected with COVID-19 (87.2%–92.8%). This proportion dropped to 19.3%–42.0% when the current situations were considered, when institutionalized interventions were in place and personal protective measures were conducted en masse (Appendix Table 1).

Perceived severity decreased significantly (p<0.001) over the study period, from 97.4% (R1) of respondents considering COVID-19 to be serious to 77.2% (R5). The perceived chance of having COVID-19 cured increased significantly (p<0.001) by more than 3-fold, from 16.6% (R1) to 57.2% (R5). An increasing time trend (p<0.001) was also observed for perceived survival chance if infected, from 18.6% (R1) to 67.2% (R5).

### Psychological Distress

The mean Hospital, Anxiety and Depression Scale—Anxiety score remained borderline abnormal throughout the study, ranging from 8.99 (R1) to 7.61 (R5). There was a substantial increase in the proportion of normal respondents in terms of anxiety (p<0.001), from 35.6% (R1) to 51.7% (R5) (Appendix Table 6). This anxiety metric echoed the significant drop in the frequency of thinking about COVID-19 (p<0.001), from 76.2% (R1) to 48.6% (R5). Despite this ease in anxiety level, the proportion of respondents worrying specifically about COVID-19 (85.7%–96.8%) and having their daily lives affected a lot by COVID-19 (45.7%–61.8%) remained high throughout the study (Appendix Table 6). Meanwhile, ≈40% of the respondents have shown symptoms of burnout toward COVID-19 since R3.

### Adoption of Precautionary Measures

The adoption of individual precautionary measures remained high throughout the study (Appendix Table 1). Most respondents reported they wore masks (R1, 99.0%; R5, 99.8%), covered mouth and nose when coughing or sneezing (R1, 96.9%; R5, 98.4%), performed hand hygiene using hand sanitizer or alcohol gel (R1, 95.8%; R5, 97.7%), and disinfected their homes (R1, 78.6%; R5, 88.5%). Hand hygiene and home disinfection measures showed a significant increasing trend across time.

The adoptions of social distancing across rounds were consistently from moderate to high (Appendix Table 1). About one third of respondents avoided public transportation (R1, 38.0% to R5, 35.6%; p = 0.11) and work (R1, 24.6% to R5, 35.4%; p<0.001) across waves. Upward significant trends were observed among respondents in avoiding social activities (R1, 63.8% to R5, 85.7%; p<0.001) and contacting with persons with fever or symptoms of respiratory diseases (R1, 92.9% to R5, 95.1%; p<0.05).

Factors associated with greater adoption of social-distancing were being female (aOR 1.30, 95% CI 1.09–1.56); living in the New Territories, a suburb of Hong Kong (aOR for the 2 territories 1.40–1.42); and being anxious (aOR 1.47, 95% CI 1.23–1.76) (Appendix Table 7). Respondents with chronic conditions (aOR 0.72, 95% CI 0.54–0.95) and those reporting having neutral understanding of COVID-19, compared with those who said they understood COVID-19 not well/not well at all (aOR 0.73, 95% CI 0.62–0.85), were less likely to practice social distancing (Appendix Table 7).

### Likelihood of COVID-19 Vaccine Uptake

Respondents’ intention to receive COVID-19 vaccine decreased significantly from R4 (48.7%, 95% CI 44.0–53.4) to R5 (37.6%, 95% CI 32.9–42.4), with particularly low rates among persons >55 years of age (Appendix Table 8). Factors associated with higher tendency for receiving COVID-19 vaccines were anxiety (borderline abnormal: aOR 1.53, 95% CI 1.04–2.23; abnormal: aOR 1.87, 95% CI 1.19–2.93), complacency (aOR 0.72, 95% CI 0.62–0.85), confidence (aOR 1.71, 95% CI 1.48–1.99), and collective responsibility (aOR 1.31, 95% CI 1.10–1.55). Compared with persons 18–24 years of age, persons >55 years of age were less likely to receive COVID-19 vaccine (aOR 0.47, 95% CI 0.23–0.98) (Appendix Table 8).

We also researched the trends of other psychobehavioral responses. We compiled responses for comparing perceived severity across diseases (Appendix Table 9), confidence in government and doctors (Appendix Table 10), knowledge of COVID-19 (Appendix Table 11), and perceived effectiveness of precautionary measures (Appendix Table 2).

## Discussion

Our 5-round longitudinal online survey analyzed the temporal changes in community responses throughout the first 2 COVID-19 epidemics in Hong Kong. Overall, perceived susceptibility (assuming no precautionary measure taken) remained high: self-susceptibility (87.2%–92.8%) was substantially higher than that observed for the 2003 SARS epidemic (23.0%) (*21*) and the 2009 influenza pandemic (58.1%) (*22*) in the same population. However, in terms of perceived severity, the proportions dropped dramatically across time but were still higher than those observed in other highly affected locations (United Kingdom, 20.7% [*12*]; Kerala state, India, 55.7% [*23*]). The proportions of persons with an abnormal level of anxiety also dropped over the study period, from 34.3% to 22.0%. We observed consistently high levels of precautionary measures, such as mask wearing, hand hygiene, and home disinfection throughout the study period. Greater anxiety was associated with higher tendency of social distancing. The projected COVID-19 vaccine uptake rate dropped from 48.7% (R4) to 37.6% (R5). Greater anxiety, confidence in the vaccine, and collective responsibility and lower complacency contributed to a greater likelihood of intended vaccination.

### Implications of Results

Our results have 5 immediate public health implications. First, with the uncertain disease progression (e.g., emergence of new variants of coronavirus) and the changing institutionalized interventions, there should be continual monitoring of risk perception toward COVID-19 in the community. Risk perception is an indispensable determinant of behavioral change (*24*) and depends on the prevalence of the health risk concerned (*25*). Our findings show time-varying risk perception over time during the pandemic, illustrating a perceived severity of COVID-19 that significantly decreased over time. Inferring from the large difference between naive (assuming no precautionary measures) and current (based on the current situation) scenarios, perceived susceptibility is sensitive to the disease progression and interventions in place. Although such temporal trend of risk perception was also observed in past pandemics (*26*), the absolute level of risk perception was not.

Second, surveillance and encouragement of social distancing should be maintained in the medium to long term, given the low projected uptake rate of COVID-19 vaccine. In Hong Kong, the reproductive number peaked at 2.39 in wave 1 and 3.04 in wave 2 (*20*), which (based on early data) corresponded approximately to requiring 56.1%–66.9% of the population to be immune to confer herd immunity (*27*). Because the projected vaccine uptake rates (R4, 48.7%; R5, 37.6%) fell short of the required level, relatively small-scale upcoming epidemics compared with the previous waves are expected. With more persons being vaccinated, there might be more social interactions, should be encouraged to maintain social distancing (such as avoiding unnecessary gatherings). Meanwhile, further research should focus on disease transmission during a mix of social distancing in place and vaccine hesitancy in the population.

Third, risk communications in Hong Kong should target complacency, vaccine confidence, and collective responsibility to boost the COVID-19 vaccine uptake. We reported a low intention for uptake of the would-be vaccines, which declined over time in Hong Kong. A similar situation was observed in the United States, where the projected vaccine uptake rate dropped from 74.1% in April 2020 to 56.2% in December 2020 (*28*). Such low uptake intention among older persons in our study (R4, 29.4%; R5, 31.4%) is particularly worrisome because older age is a risk factor for death from COVID-19 (*29*).

The extent to which our findings on the predictors of uptake intention can be generalized to other countries or regions requires further investigation. Unique to Hong Kong were the low COVID-19 infection rate and low level of confidence in government measures. The weak uptake intention reported in this study was uncommon compared with other countries (71.5% overall for 19 countries) (*30*). The low infection rate, along with the decreasing perceived severity toward COVID-19, might weaken the urgency for vaccination, which may also apply to places such as Taiwan, Japan, and Australia. However, the social unrest in Hong Kong in late 2019 might have led to distrust in the government (*31*), which could subsequently lower vaccination intention (*32*) and trigger maintenance of personal precautionary measures. One possible explanation is that, when moderated by distrust in government, persons tend to rely on personal protective measures (such as wearing facemasks and maintaining social distancing) but become skeptical to institutional protective measures (such as vaccines). Distrust in governments during the pandemic may also influence vaccine hesitancy in other regions, such as Brazil and Poland (*33*). Nevertheless, given the projected low vaccine uptake rate in this study, it may be insufficient to reach herd immunity in the near future, if ever, in Hong Kong. Therefore, taking the vaccine or not may have little bearing on relaxing government interventions in the medium term. In addition, from findings in other regions, trust in the government itself (*34*) and the information provided by the government (*30*) increased preventive practices, specifically accepting vaccines, during pandemics (*30*). Therefore, effective health communication is particularly crucial for the Hong Kong government. To rebuild trust, public health authorities need to possess competence, objectivity, fairness, consistency, transparency, sincerity, and faith (*35*). In addition, organizations aside from government and healthcare providers, such as professional bodies and religious groups, may help deliver pro-vaccine messages (*36*).

Fourth, our results help to prioritize the content in promotional messaging. It is worth investing resources on promotional messaging, particularly when few respondents in R4 (overall, 16.7%; 18–24 y, 24.7%; 25–34 y, 14.5%; 35–44 y, 15.5%; 45–54 y, 11.5%; >55 y, 17.6%) and R5 (overall, 10.5%; 18–24 y, 12.8%; 25–34 y, 7.4%; 35–44 y, 12.1%; 45–54 y, 6.1%; >55 y, 20.0%) indicated an absolute “yes” for receiving COVID-19 vaccines (measured on a 11-point Likert scale) and when there was antibody waning after receiving the vaccine. The decreasing confidence metric from R4 to R5 highlighted the need to build trust among the public toward the logistics of vaccine development, licensing, generating recommendations, and distribution (*37*). Before the government authorizes the use of a COVID-19 vaccine, establishment of an advisory panel will help determine factors that the government should consider, such as performance (safety, efficacy, and effectiveness) and characteristics (number of doses, formulation, and presentation and packaging) of the available vaccine (*38*). Moreover, to increase the collective responsibility and perceived vaccine necessity, the government should foster understanding of the vaccine among the public with transparent communication, including more engagement with different stakeholders in the community and populations who are disproportionately affected by the pandemic to listen to their concerns. Leveraging knowledge, skills, and expertise from these communications will provide a robust assessment to underpin the vaccination campaign. Although calculations and constraints in the 5Cs model were not associated with the vaccine uptake likelihood at this stage, continuous examination in these 2 constructs will help refine future vaccination campaigns to engage citizens in cost–benefit calculations and increase their vaccine availability, affordability and accessibility.

Fifth, the psychological distress arising from burnout should be weighed together with the well-established anxiety. This pandemic is ongoing and has lasted much longer than the SARS epidemic, so more persons are developing syndromes of emotional exhaustion. The interplay between 2 psychological distresses, burnout and anxiety, is worth investigating during the ongoing pandemic. Our study showed that almost half of respondents had burnout symptoms in a short 4-month window from June through September 2020. This symptom did not contribute to the likelihood of COVID-19 vaccination in the last 2-point survey. However, the current general measure of burnout was not able to pinpoint the sources of burnout, such as financial stress, social isolation, the disease itself, or their combinations, for a detailed analysis. Nevertheless, the burnout phenomenon among persons coping with a long-term pandemic (*39*) suggests the need to reexamine the temporal association among social-distancing adoption, vaccination, and burnout.

Our study’s first limitation is that the survey may have been subject to recall and social conformity biases, but its longitudinal design enabled us to track the same respondents over time, reducing self-control bias. Second, caution should be exercised when generalizing our findings to other regions because Hong Kong was exposed to other disease outbreaks recently, such as 1997 avian influenza **(***40*), 2003 SARS (*41*), and 2009 pandemic influenza (*42*). Nevertheless, our COVID-19 experience after those past outbreaks may be precedent to other countries, after the current COVID-19 pandemic. Third, our survey was conducted before the safety and efficacy data of the COVID-19 vaccines were released. The actual uptake rates might be affected by possible vaccination side effects events, such as the recent reported deaths after taking the vaccines in Hong Kong (*43**–**45*).

In conclusion, our findings highlight the importance of continuous longitudinal assessment of community psychobehavioral responses during the COVID-19 pandemic. Monitoring those responses can help public health authorities tailor health communication strategies to achieve the desired behavioral outcomes (vaccination and adoption of precautionary measures) to control future epidemic waves.

Appendix Additional information on psychobehavioral responses and likelihood of receiving COVID-19 vaccines during the pandemic, Hong Kong.
