# Supplementary material for: Psychobehavioral Responses and Likelihood of Receiving COVID-19 Vaccines during the Pandemic, Hong Kong
Source: Emerg Infect Dis. 2021 Jul;27(7):1802–10. doi: 10.3201/eid2707.210054 (PMC8237883; doi:10.3201/eid2707.210054)
Supplement: Appendix — Additional information on psychobehavioral responses and likelihood of receiving COVID-19 vaccines during the pandemic, Hong Kong. [file 21-0054-Techapp-s1.pdf]

Article DOI: https://doi.org/10.3201/eid2707.210054

Psychobehavioral Responses and Likelihood of Receiving COVID-19 Vaccines during the Pandemic, Hong Kong

Appendix

Additional Demographic Details of Study Cohort

In the 14 days before the survey, the proportion of subjects attending medical consultation remained at 15.1%–18.1%, but the proportion of respondents who had respiratory symptoms dropped by almost half, from 23.9% (R1) to 12.6% (R5) (Appendix Table 4). Since R1, the proportion of subjects paying an outbound visit has plummeted, from 23.8% (R1) to 0% (R5) (Appendix Table 4).

Comparison of Perceived Severity across Noncommunicable Diseases and Infectious Diseases

The perceived severity of COVID-19 was also delineated together with other diseases (Appendix Table 9). In general, participants consistently regarded COVID-19 (95.5%–99.5%) as being as serious as 2003 severe acute respiratory syndrome (97.4%–99.2%), acquired immunodeficiency syndrome (93.8%–97.5%), heart diseases (94.2%–98.1%), and cancer (95.9%–98.8%). It stood out from the annually circulating seasonal influenza (54.4%–66.6%) and the 2009 pandemic influenza (78.4%–87.7%).

Confidence in Government and Doctors over Time

Participants' confidence in the local government's adopted measures, ability and decisiveness to deal with COVID-19, and, in general, the ability to manage major crises remained relatively low during the epidemic periods (Appendix Table 10). The proportion of participants who were confident in the COVID-19 information provided by the local government

varied significantly ($p<0.001$), ranging from 6.5% to 13.5%. On the contrary, subjects' confidence in doctors' ability to diagnose COVID-19 remained high, and increased substantially, from 53.3% (R1) to 69.6% (R5) ($p<0.001$).

Knowledge about COVID-19 over Time

Almost all respondents paid continued attention to the COVID-19 progress throughout the first 2 waves of epidemics in Hong Kong (96.6%–99.4%), but the proportion of subjects who took the initiative to search for COVID-19 information dropped from the initial 83.2% (R1) to as low as 64.1% (R3) during the quiescence between 2 epidemic waves (Appendix Table 11). As time passed, the proportion of respondents who regarded themselves as not understanding COVID-19 well increased from the initial 64.8% (R1) to 94.9% (R5) (Appendix Table 11).

Over time, decreasing trends were observed in the percentage of respondents who identified eating game (wild animals) (R1, 92.6% to R5, 73.4%; $p<0.001$), going to wet markets (R1, 80.8% to R5, 72.7%; $p<0.001$), eating seafood imported from Wuhan (R1, 72.4% to R5, 55.1%; $p<0.001$), and eating other products from Wuhan (R1, 66.5% to R5, 51.2%; $p<0.001$) as likely routes of transmission of COVID-19. The proportion of respondents who regarded droplets as a likely route of transmission remained high (R1, 98.5% to R5, 98.1%) throughout the study but the trend was not found to be significant ($p = 0.45$). A significant increase in the number of respondents to recognize aerosol as a route of transmission was found (R1, 88.6% to R5, 90.9%; $p = 0.01$) (Appendix Table 11).

Perceived Effectiveness of Precautionary Measures

Almost all participants (>90%) felt that wearing masks, washing hands frequently with hand sanitizer or alcohol gel, disinfecting homes, covering mouth and nose when coughing and sneezing, avoiding contact with persons with respiratory disease symptoms, and avoiding going to crowded places were effective or very effective precautionary measures (Appendix Table 2) throughout the study. Relatively smaller proportions of respondents considered avoiding public transportation (R1, 76.1% to R5, 78.9%, with no significant time trend [$p = 0.28$]) and the wet market (R1, 69.9% to R5, 79.2%, with an increasing trend across waves [$p<0.001$]) were very effective or effective measures (Appendix Table 2).

Appendix Table 1. Adoption of precautionary measures, Hong Kong, 2020

Precautionary measures	Round 1 (N = 2,478) n	%	Round 2 (N = 644) n	%	Round 3 (N = 542) n	%	Round 4 (N = 484) n	%	Round 5 (N = 441) n	%	p (trend)
Personal hygiene											
Wear mask											0.09
Yes	2,095	99.0	619	99.4	527	99.6	461	99.1	426	99.8	
No/not applicable	21	1.0	4	0.6	2	0.4	4	0.9	1	0.2	
Wash hands frequently (use hand sanitizer/alcohol gel)											0.03
Yes	2,027	95.8	614	98.6	522	98.7	452	97.2	417	97.7	
No/not applicable	89	4.2	9	1.4	7	1.3	13	2.8	10	2.3	
Disinfect home											0.00
Yes	1,663	78.6	560	89.9	469	88.7	411	88.4	378	88.5	
No/not applicable	453	21.4	63	10.1	60	11.3	54	11.6	49	11.5	
Cover mouth and nose when coughing or sneezing											0.13
Yes	2,051	96.9	612	98.2	519	98.1	451	97.0	420	98.4	
No/not applicable	65	3.1	11	1.8	10	1.9	14	3.0	7	1.6	
Social distancing											
Avoid contact with persons with fever or symptoms of respiratory diseases											0.05
Yes	1,965	92.9	597	95.8	497	94.0	438	94.2	406	95.1	
No/not applicable	151	7.1	26	4.2	32	6.0	27	5.8	21	4.9	
Avoid contact with persons who have been to Wuhan in the past month											0.00
Yes	1,781	84.2	544	87.3	423	80.0	361	77.6	308	72.1	
No/not applicable	335	15.8	79	12.7	106	20.0	104	22.4	119	27.9	
Avoid going out											0.00
Yes	1445	68.3	494	79.3	389	73.5	360	77.4	322	75.4	
No/not applicable	671	31.7	129	20.7	140	26.5	105	22.6	105	24.6	
Avoid going to crowded places											0.40
Yes	1,845	87.2	571	91.7	460	87.0	425	91.4	372	87.1	
No/not applicable	271	12.8	52	8.3	69	13.0	40	8.6	55	12.9	
Avoid going to wet market											0.08
Yes	1,456	68.8	396	63.6	290	54.8	307	66.0	284	66.5	
No/not applicable	660	31.2	227	36.4	239	45.2	158	34.0	143	33.5	
Avoid going to hospital/clinic											0.00
Yes	1,605	75.9	503	80.7	373	70.5	334	71.8	294	68.9	
No/not applicable	511	24.1	120	19.3	156	29.5	131	28.2	133	31.1	
Avoid public transportation											0.11
Yes	805	38.0	272	43.7	168	31.8	169	36.3	152	35.6	
No/not applicable	1,311	62.0	351	56.3	361	68.2	296	63.7	275	64.4	
Avoid work											0.00
Yes	521	24.6	238	38.2	139	26.3	162	34.8	151	35.4	
No/not applicable	1,595	75.4	385	61.8	390	73.7	303	65.2	276	64.6	
Avoid social activities											0.00
Yes	1,350	63.8	519	83.3	395	74.7	400	86.0	366	85.7	
No/not applicable	766	36.2	104	16.7	134	25.3	65	14.0	61	14.3	
Travel avoidance											
Avoid going to Wuhan											0.00
Yes	1,895	89.6	538	86.4	406	76.7	335	72.0	288	67.4	
No/not applicable	221	10.4	85	13.6	123	23.3	130	28.0	139	32.6	
Avoid going to Hubei province											0.00

Precautionary measures	Round 1 (N = 2,478) n	Round 1 %	Round 2 (N = 644) n	Round 2 %	Round 3 (N = 542) n	Round 3 %	Round 4 (N = 484) n	Round 4 %	Round 5 (N = 441) n	Round 5 %	p (trend)
Yes	1,894	89.5	538	86.4	406	76.7	338	72.7	288	67.4	
No/not applicable	222	10.5	85	13.6	123	23.3	127	27.3	139	32.6	
Avoid going to mainland China											0.00
Yes	1,942	91.8	566	90.9	431	81.5	363	78.1	314	73.5	
No/not applicable	174	8.2	57	9.1	98	18.5	102	21.9	113	26.5	
Leave Hong Kong temporarily											0.08
Yes	205	9.7	53	8.5	44	8.3	54	11.6	56	13.1	
No/not applicable	1,911	90.3	570	91.5	485	91.7	441	88.4	371	86.9	

Appendix Table 2. Perceived effectiveness of precautionary measures to prevent COVID-19, Hong Kong, 2020

Precautionary measures	Round 1 (N = 2,478) n	Round 1 %	Round 2 (N = 644) n	Round 2 %	Round 3 (N = 542) n	Round 3 %	Round 4 (N = 484) n	Round 4 %	Round 5 (N = 441) n	Round 5 %	p (trend)
Wear mask											0.00
Very effective/effective	1,988	94.0	604	97.0	521	98.5	459	98.7	422	98.8	
Neutral/not effective/not very effective	128	6.0	19	3.0	8	1.5	6	1.3	5	1.2	
Wash hands frequently (use hand sanitizer/alcohol gel)											0.00
Very effective/effective	2,038	96.3	611	98.1	525	99.2	462	99.4	425	99.5	
Neutral/not effective/not very effective	78	3.7	12	1.9	4	0.8	3	0.6	2	0.5	
Disinfect home											0.00
Very effective/effective	1,910	90.3	595	95.5	512	96.8	448	96.3	411	96.3	
Neutral/not effective/not very effective	206	9.7	28	4.5	17	3.2	17	3.7	16	3.7	
Cover mouth and nose when coughing or sneezing											0.00
Very effective/effective	1,917	90.6	597	95.8	509	96.2	454	97.6	412	96.5	
Neutral/not effective/not very effective	199	9.4	26	4.2	20	3.8	11	2.4	15	3.5	
Avoid contact with persons with fever or symptoms of respiratory diseases											0.00
Very effective/effective	2,045	96.6	616	98.9	522	98.7	460	98.9	421	98.6	
Neutral/not effective/not very effective	71	3.4	7	1.1	7	1.3	5	1.1	6	1.4	
Avoid contact with persons who have been to Wuhan in the past month											0.00
Very effective/effective	2,047	96.7	610	97.9	502	94.9	415	89.2	378	88.5	
Neutral/not effective/not very effective	69	3.3	13	2.1	27	5.1	50	10.8	49	11.5	
Avoid going out											0.00
Very effective/effective	1,720	81.3	545	87.5	442	83.6	425	91.4	381	89.2	
Neutral/not effective/not very effective	396	18.7	78	12.5	87	16.4	40	8.6	46	10.8	
Avoid going to crowded places											0.00
Very effective/effective	1,943	91.8	603	96.8	491	92.8	448	96.3	411	96.3	
Neutral/not effective/not very effective	173	8.2	20	3.2	38	7.2	17	3.7	16	3.7	
Avoid going to wet market											0.00
Very effective/effective	1,479	69.9	418	67.1	326	61.6	353	75.9	338	79.2	
Neutral/not effective/not very effective	637	30.1	205	32.9	203	38.4	112	24.1	89	20.8	
Avoid going to hospital/clinic											0.01
Very effective/effective	1,846	87.2	542	87.0	418	79.0	395	84.9	356	83.4	
Neutral/not effective/not very effective	270	12.8	81	13.0	111	21.0	70	15.1	71	16.6	
Avoid public transportation											0.28
Very effective/effective	1,610	76.1	506	81.2	375	70.9	370	79.6	337	78.9	
Neutral/not effective/not very effective	506	23.9	117	18.8	154	29.1	95	20.4	90	21.1	

Precautionary measures	Round 1 (N = 2,478) n	%	Round 2 (N = 644) n	%	Round 3 (N = 542) n	%	Round 4 (N = 484) n	%	Round 5 (N = 441) n	%	p (trend)
Avoid work											0.00
Very effective/effective	1,453	68.7	485	77.8	368	69.6	379	81.5	338	79.2	
Neutral/not effective/not very effective	663	31.3	138	22.2	161	30.4	86	18.5	89	20.8	
Avoid social activities											0.00
Very effective/effective	1,602	75.7	560	89.9	438	82.8	432	92.9	389	91.1	
Neutral/not effective/not very effective	514	24.3	63	10.1	91	17.2	33	7.1	38	8.9	
Avoid going to Wuhan											0.00
Very effective/effective	2,070	97.8	612	98.2	505	95.5	426	91.6	378	88.5	
Neutral/not effective/not very effective	46	2.2	11	1.8	24	4.5	39	8.4	49	11.5	
Avoid going to Hubei province											0.00
Very effective/effective	2,075	98.1	613	98.4	506	95.7	422	90.8	379	88.8	
Neutral/not effective/not very effective	41	1.9	10	1.6	23	4.3	43	9.2	48	11.2	
Avoid going to mainland China											0.00
Very effective/effective	2,057	97.2	612	98.2	504	95.3	429	92.3	382	89.5	
Neutral/not effective/not very effective	59	2.8	11	1.8	25	4.7	36	7.7	45	10.5	
Leave Hong Kong temporarily											0.00
Very effective/effective	618	29.2	88	14.1	75	14.2	90	19.4	92	21.5	
Neutral/not effective/not very effective	1,498	70.8	535	85.9	454	85.8	375	80.6	335	78.5	

Appendix Table 3. Demographic characteristics of study participants, Hong Kong, 2020

Demographic characteristic*	Round 1 (N = 2,478) n	%	Round 2 (N = 644) n	%	Round 3 (N = 542) n	%	Round 4 (N = 484) n	%	Round 5 (N = 441) n	%
Sex										
Male	734	31.5	198	30.7	167	30.8	146	30.2	139	31.5
Female	1,594	68.5	446	69.3	375	69.2	338	69.8	302	68.5
Age, y										
18–24	588	25.3	131	20.3	107	19.7	100	20.7	90	20.4
25–34	750	32.2	220	34.2	188	34.7	172	35.5	158	35.8
35–44	513	22.0	156	24.2	131	24.2	120	24.8	105	23.8
45–54	263	11.3	83	12.9	66	12.2	54	11.2	52	11.8
≥55	214	9.2	54	8.4	50	9.2	38	7.9	36	8.2
Educational attainment										
Junior high school or below	58	3.4	15	2.3	9	1.7	7	1.4	9	2.0
High school	305	17.7	107	16.6	86	15.9	80	16.5	68	15.4
Postsecondary	1,136	66.0	418	64.9	351	64.8	326	67.4	294	66.7
Graduate school or above	221	12.8	104	16.1	96	17.7	71	14.7	70	15.9
Presence of domestic helper at home										
Yes	209	12.2	87	13.5	72	13.3	62	12.8	59	13.4
No	1,507	87.8	557	86.5	470	86.7	422	87.2	382	86.6
Presence of children at home										

Demographic characteristic*	Round 1 (N = 2,478) n	%	Round 2 (N = 644) n	%	Round 3 (N = 542) n	%	Round 4 (N = 484) n	%	Round 5 (N = 441) n	%
Yes	462	26.9	192	29.8	164	30.3	145	30.0	140	31.7
No	1,254	73.1	452	70.2	378	69.7	339	70.0	301	68.3
Total monthly household income (Hong Kong dollars [HKD])										
<u><</u>10,000 or below	104	6.1	25	3.9	24	4.4	19	3.9	18	4.1
10,001–20,000†	277	16.2	95	14.8	68	12.5	68	14.0	65	14.7
20,001–30,000	297	17.3	125	19.4	100	18.5	85	17.6	84	19.0
30,001–40,000	233	13.6	97	15.1	89	16.4	87	18,0	72	16.3
40,001–50,000	162	9.4	61	9.5	56	10.3	50	10.3	45	10.2
50,001–60,000	128	7.5	57	8.9	47	8.7	41	8.5	42	9.5
>60,000	257	15.0	118	18.3	102	18.8	86	17.8	77	17.5
Not disclosed	257	15.0	66	10.2	56	10.3	48	10.0	38	8.6
District of residence										
Hong Kong Island	400	17.2	101	15.8	87	16.1	73	15.2	76	17.4
Kowloon East	392	16.8	111	17.3	79	14.6	85	17.7	72	16.4
Kowloon West	168	7.2	52	8.1	46	8.5	38	7.9	33	7.5
New Territories East	734	31.5	201	31.4	171	31.6	150	31.3	138	31.5
New Territories West	633	27.2	175	27.3	159	29.3	134	27.9	119	27.2

*Demographic data for rounds 2–5 were retrieved from data in round 1, except for district of residence.
†In the survey this option was shown as "HKD 10,000–HKD 20,000."

Appendix Table 4. Background health conditions and travel history of participants, Hong Kong, 2020

Condition	Round 1 (N = 2,478) n	%	Round 2 (N = 644) n	%	Round 3 (N = 542) n	%	Round 4 (N = 484) n	%	Round 5 (N = 441) n	%
Background health conditions										
Present smoker*										
Yes†	217	8.9	54	8.4	40	7.4	41	8.5	36	8.2
No‡	2,211	91.1	590	91.6	502	92.6	443	91.5	405	91.8
Presence of chronic disease*										
Yes	192	11.2	83	12.9	66	12.2	58	12.0	51	11.6
No	1,523	88.8	561	87.1	476	87.8	426	88.0	390	88.4
Self-perceived health condition										
Excellent/good	1,870	77.0	499	78.1	418	77.3	350	73.1	335	76.8
Bad/very bad	517	21.3	129	20.2	109	20.1	113	23.6	88	20.2
Fair	41	1.7	11	1.7	14	2.6	16	3.3	13	3.0
Medical consultation in past 14 d										
Yes	418	17.2	109	17.1	98	18.1	86	18.0	66	15.1
No	2,010	82.8	530	82.9	443	81.9	393	82.0	370	84.9
Presence of respiratory symptom(s) in past 14 d										
Yes	581	23.9	115	18.0	58	10.7	77	16.1	55	12.6

Condition	Round 1 (N = 2,478) n	%	Round 2 (N = 644) n	%	Round 3 (N = 542) n	%	Round 4 (N = 484) n	%	Round 5 (N = 441) n	%
No	1,847	76.1	524	82.0	483	89.3	402	83.9	381	87.4
Travel history										
Regular visits to mainland China*										
Yes	72	3.0	21	3.3	20	3.7	18	3.7	13	2.9
No	2,346	97.0	623	96.7	522	96.3	466	96.3	428	97.1
Leave Hong Kong in past month										
Yes	575	23.8	40	6.3	2	0.4	2	0.4	0	0
No	1,844	76.2	598	93.7	538	99.6	477	99.6	434	100

*Demographic data for rounds 2–5 were retrieved from data in round 1, except for district of residence.
†Regular and social smoker.
‡Nonsmoker (quit smoking for >1 mo) and never-smoker.

Appendix Table 5. Risk perception toward COVID-19, Hong Kong, 2020

Condition	Round 1 (N = 2,478) n	%	Round 2 (N = 644) n	%	Round 3 (N = 542) n	%	Round 4 (N = 484) n	%	Round 5 (N = 441) n	%	p (trend)
Perceived susceptibility (assuming no precautionary measures)											
Self											0.03
Very likely/likely	2,039	87.2	579	91.3	470	87.9	437	92.8	384	89.3	
Neutral/unlikely/very unlikely	298	12.8	55	8.7	65	12.1	34	7.2	46	10.7	
Family members											0.08
Very likely/likely	2,122	90.8	600	94.6	487	91.0	446	94.7	397	92.3	
Neutral/unlikely/very unlikely	215	9.2	34	5.4	48	9.0	25	5.3	33	7.7	
Perceived susceptibility (based on current situation)											
Self											0.00
Very high/high	NA*	NA	NA	NA	103	19.3	198	42.0	144	33.5	
Neutral/small/very small	NA	NA	NA	NA	432	80.7	273	58.0	286	66.5	
Family members											0.00
Very high/high	NA	NA	NA	NA	104	19.4	217	46.1	153	35.6	
Neutral/small/very small	NA	NA	NA	NA	431	80.6	254	53.9	277	64.4	
Perceived severity											
Perceived severity of COVID-19											0.00
Very serious/serious	2,266	97.4	595	93.8	471	88.0	387	82.2	332	77.2	
Neutral/not serious/not serious at all	61	2.6	39	6.2	64	12.0	84	17.8	98	22.8	
Perceived chance of having COVID-19 cured											0.00
Very high/high	386	16.6	228	36.0	239	44.7	257	54.6	246	57.2	
Neutral/low/very low	1,941	83.4	406	64.0	296	55.3	214	45.4	184	42.8	
Perceived survival chance of COVID-19											0.00
Very high/high	432	18.6	289	45.6	309	57.8	310	65.8	289	67.2	
Neutral/low/very low	1,895	81.4	345	54.4	226	42.2	161	34.2	141	32.8	

*NA, not applicable.

Appendix Table 6. Worry and burnout of participants, Hong Kong, 2020*

Characteristic	Round 1 (N = 2,478) n	%	Round 2 (N = 644) n	%	Round 3 (N = 542) n	%	Round 4 (N = 484) n	%	Round 5 (N = 441) n	%	p (trend)
Anxiety (general)											
Anxiety level by HADS-A											0.00
Normal	850	35.6	233	36.6	264	49.1	209	44.2	223	51.7	
Borderline	719	30.1	201	31.6	142	26.4	136	28.8	113	26.2	
Abnormal	819	34.3	202	31.8	132	24.5	128	27.1	95	22.0	
Anxiety (COVID-19 specific)											
Effect of COVID-19 on daily life											0.57
A little/not at all/do not know	1,074	44.5	254	39.8	292	54.3	182	38.2	182	42.0	
A lot	1,338	55.5	384	60.2	246	45.7	294	61.8	251	58.0	
Worried about COVID-19											0.00
Yes	2,335	96.8	597	93.6	461	85.7	440	92.4	377	87.1	
No	77	3.2	41	6.4	77	14.3	36	7.6	56	12.9	
Fear of contracting COVID-19 in public places											0.00
Very afraid/afraid/neutral	2,351	97.8	622	97.7	503	93.5	453	95.2	406	94.0	
Not afraid/not afraid at all	53	2.2	15	2.4	35	6.5	23	4.8	26	6.0	
Frequency of thinking of COVID-19											0.00
Always (could not fall asleep)/very often/often	1,831	76.2	431	67.7	262	48.7	285	59.9	210	48.6	
Sometimes/never	573	23.8	206	32.3	276	51.3	191	40.1	222	51.4	
Burnout											
Presence of burnout symptoms											0.53
Yes	NA	NA	NA	NA	195	37.6	180	39.6	168	40.1	
No	NA	NA	NA	NA	324	62.4	274	60.4	251	59.9	

*HADS-A, Hospital, Anxiety, and Depression Scale--Anxiety; NA, not applicable.

Appendix Table 7. Proportion of participants with higher tendency of social distancing, and factors associated with such higher tendency, Hong Kong, 2020

	Round 1		Round 2		Round 3		Round 4		Round 5			Higher tendency of social distancing		
		Proportion		Proportion		Proportion		Proportion		Proportion	p			
Characteristic	n	% (95% CI)	n	% (95% CI)	n	% (95% CI)	n	% (95% CI)	n	% (95% CI)	(trend)	aOR	(95% CI)	p value
Overall	1,715	48.5 (46.1–50.9)	623	55.7 (51.7–59.6)	529	45.6 (41.3–49.9)	465	52.3 (47.6–56.9)	427	52.7 (47.8–57.5)	0.09	NA	NA	NA
Sex														
Male	539	43.4 (39.2–47.7)	190	51.1 (43.7–58.3)	161	37.3 (29.9–45.3)	140	50.7 (42.2–59.2)	133	50.4 (41.6–59.1)	0.06	NA	NA	NA
Female	1176	50.9 (48.0–53.7)	433	57.7 (52.9–62.4)	368	49.2 (44.0–54.4)	325	52.9 (47.3–58.4)	294	53.7 (47.9–59.5)	0.47	1.30	(1.09–1.56)	0.00
Age, y														
18–24	441	46.5 (41.8–51.3)	124	65.3 (56.2–73.5)	106	47.2 (37.5–57.1)	96	51.0 (40.7–61.3)	86	58.1 (47.0–68.5)	0.05	NA	NA	NA
25–34	558	52.3 (48.1–56.5)	214	55.1 (48.2–61.9)	184	47.8 (40.5–55.3)	168	54.2 (46.3–61.8)	150	56.0 (47.7–64.0)	0.24	1.18	(0.94–1.47)	0.15
35–44	381	48.0 (42.9–53.2)	153	55.6 (47.3–63.5)	126	50.0 (41.4–58.6)	112	58.0 (48.3–67.2)	103	46.6 (36.8–56.7)	0.51	1.11	(0.88–1.42)	0.38
45–54	197	49.2 (42.1–56.4)	79	45.6 (34.5–57.1)	65	35.4 (24.2–48.3)	53	47.2 (33.5–61.2)	52	50.0 (36.9–63.1)	0.48	1.06	(0.78–1.42)	0.72
55 or above	138	39.9 (31.7–48.6)	53	50.9 (37.0–64.7)	48	35.4 (22.6–50.6)	36	36.1 (21.3–53.8)	36	47.2 (30.8–64.3)	0.97	0.82	(0.57–1.17)	0.26
Living district														
Hong Kong Island	307	42.0 (36.5–47.8)	96	54.2 (43.7–64.3)	85	36.5 (26.5–47.7)	72	38.9 (27.8–51.1)	73	49.3 (37.5–61.2)	0.26	NA	NA	NA
Kowloon East	268	42.5 (36.6–48.7)	107	53.3 (43.4–62.9)	78	43.6 (32.6–55.3)	83	60.2 (48.9–70.6)	70	50.0 (38.6–61.4)	0.07	1.01	(0.76–1.33)	0.96
Kowloon West	128	41.4 (32.9–50.5)	52	59.6 (45.1–72.7)	45	55.6 (40.1–70.0)	37	56.8 (39.6–72.5)	32	50.0 (33.6–66.4)	0.05	1.20	(0.85–1.68)	0.31
New Territories East	541	54.3 (50.0–58.6)	196	57.1 (49.9–64.1)	167	44.9 (37.3–52.8)	145	52.4 (44.0–60.7)	136	51.5 (42.8–60.1)	0.11	1.40	(1.10–1.80)	0.01
New Territories West	471	51.4 (46.8–56.0)	172	55.2 (47.5–62.7)	154	49.4 (41.3–57.5)	128	53.1 (44.1–61.9)	116	58.6 (49.1–67.6)	0.09	1.42	(1.11–1.82)	0.01
Perceived understanding of COVID-19														
Not well/ not well at all	173	41.6 (34.3–49.4)	291	58.4 (52.5–64.1)	245	47.3 (41.0–53.8)	229	54.1 (47.5–60.7)	233	53.2 (46.6–59.7)	0.24	NA	NA	NA
Neutral	908	44.2 (40.9–47.5)	297	52.5 (46.7–58.3)	248	40.7 (34.6–47.1)	212	50.0 (43.3–56.7)	172	52.3 (44.6–59.9)	0.13	0.73	(0.62–0.85)	0.00
Well/ very well	634	56.6 (52.7–60.5)	35	60.0 (42.2–75.6)	36	66.7 (48.9–80.9)	24	54.2 (33.2–73.8)	22	50.0 (30.7–69.3)	0.85	1.02	(0.85–1.22)	0.85
Presence of chronic conditions														
No	1,523	49.2 (46.7–51.8)	544	58.1 (53.8–62.3)	463	47.7 (43.1–52.4)	407	55.0 (50.1–59.9)	377	53.6 (48.4–58.7)	0.07	NA	NA	NA
Yes	192	42.7 (35.7–50.0)	79	39.2 (28.6–50.9)	66	30.3 (19.9–43.0)	58	32.8 (21.4–46.5)	50	46.0 (32.1–60.5)	0.98	0.72	(0.54–0.95)	0.02
Anxiety level														
Normal	607	40.7 (36.8–44.7)	230	50.0 (43.6–56.4)	258	38.4 (32.5–44.6)	205	47.3 (40.4–54.4)	220	48.2 (41.4–55.0)	0.06	NA	NA	NA
Borderline abnormal	527	49.7 (45.4–54.1)	196	53.6 (46.3–60.7)	140	42.9 (34.6–51.5)	134	50.0 (41.7–58.3)	112	55.4 (45.7–64.7)	0.66	1.05	(0.89–1.23)	0.59
Abnormal	581	55.6 (51.4–59.7)	197	64.5 (57.3–71.1)	131	62.6 (53.7–70.8)	126	62.7 (53.6–71.0)	95	60.0 (49.4–69.8)	0.04	1.47	(1.23–1.76)	0.00
Confidence in government measures														
Very confident to neutral	108	36.1 (27.3–46.0)	77	59.7 (47.9–70.6)	134	38.8 (30.6–47.6)	64	53.1 (40.3–65.5)	56	53.6 (39.9–66.8)	0.11	NA	NA	NA
Not confident/ not confident at all	1,607	49.3 (46.9–51.8)	546	55.1 (50.8–59.3)	395	47.8 (42.8–52.9)	401	52.1 (47.1–57.1)	371	52.6 (47.3–57.7)	0.22	1.03	(0.84–1.27)	0.78

*aOR, adjusted odds ratio; NA, not applicable.

Appendix Table 8. Proportion of potential vaccine recipients and factors associated with higher uptake tendency of COVID-19 vaccines, Hong Kong, 2020*

	Round 4			Round 5			Temporal variation (difference in proportion)			Higher uptake tendency		
		Proportion			Proportion							
Characteristics	n	%	95% CI	n	%	95% CI	%	95% CI	p value†	aOR	95% CI	p value
Overall	454	48.7	(44.0–53.4)	418	37.6	(32.9–42.4)	−11.1	(−16.1 to −6.2)	0.00	NA	NA	NA
Sex												
Male	136	53.7	(44.9–62.2)	132	40.9	(32.5–49.8)	−12.8	(−22.0 to −3.6)	0.01	NA	NA	NA
Female	318	46.5	(41.0–52.2)	286	36.0	(30.5–41.9)	−10.5	(−16.4 to −4.7)	0.00	0.87	(0.59–1.28)	0.48
Age, y												
18–24	93	59.1	(48.4–69.1)	86	46.5	(35.8–57.5)	−12.6	(−22.6 to −2.6)	0.01	-	-	-
25–34	165	50.9	(43.1–58.7)	149	37.6	(29.9–45.9)	−13.3	(−21.7 to −4.9)	0.00	0.79	(0.48–1.30)	0.35
35–44	110	46.4	(36.9–56.1)	99	37.4	(28.0–47.7)	−9.0	(−19.1 to 1.1)	0.08	0.87	(0.51–1.48)	0.62
45–54	52	40.4	(27.3–54.9)	49	26.5	(15.4–41.3)	−13.9	(−28.5 to 0.8)	0.06	0.59	(0.29–1.21)	0.15
≥55	34	29.4	(15.7–47.7)	35	31.4	(17.4–49.4)	2.0	(−15.8 to 19.8)	0.82	0.47	(0.23–0.98)	0.04
District of residence												
Hong Kong Island	69	43.5	(31.8–55.9)	72	27.8	(18.2–39.8)	−15.7	NA	NA	NA	NA	NA
Kowloon East	82	53.7	(42.4–64.6)	70	37.1	(26.1–49.6)	−16.5	NA	NA	1.56	(0.86–2.85)	0.14
Kowloon West	36	47.2	(30.8–64.3)	32	34.4	(19.2–53.2)	−12.8	NA	NA	1.08	(0.50–2.34)	0.84
New Territories E	141	48.9	(40.5–57.5)	132	37.1	(29.0–46.0)	−11.8	NA	NA	1.26	(0.73–2.17)	0.41
New Territories W	126	48.4	(39.5–57.4)	112	45.5	(36.2–55.2)	−2.9	NA	NA	1.57	(0.90–2.75)	0.11
Perceived understanding of COVID-19												
Not well/not well at all	226	53.1	(46.4–59.7)	229	38.9	(32.6–45.5)	−14.2	NA	NA	NA	NA	NA
Neutral	204	42.2	(35.4–49.3)	168	36.9	(29.7–44.7)	−5.3	NA	NA	1.13	(0.80–1.61)	0.48
Well or very well	24	62.5	(40.8–80.4)	21	28.6	(12.2–52.3)	−33.9	NA	NA	1.11	(0.56–2.21)	0.76
Presence of chronic conditions												
No	399	48.1	(43.1–53.1)	370	36.2	(31.4–41.4)	−11.9	(−17.1 to −6.8)	0.00	NA	NA	NA
Yes	55	52.7	(38.9–66.1)	48	47.9	(33.5–62.6)	−4.8	(−21.3 to 11.7)	0.57	1.55	(0.88–2.74)	0.13
Anxiety level												
Normal	200	42.5	(35.6–49.7)	215	32.6	(26.4–39.3)	−9.9	NA	NA	NA	NA	NA
Borderline abnormal	130	51.5	(42.7–60.3)	110	41.8	(32.6–51.6)	−9.7	NA	NA	1.53	(1.04–2.23)	0.03
Abnormal	124	55.6	(46.5–64.5)	93	44.1	(33.9–54.7)	−11.6	NA	NA	1.87	(1.19–2.93)	0.01
Confidence in government measures												
Very confident/neutral	63	44.4	(32.1–57.4)	55	40.0	(27.3–54.1)	−4.4	NA	NA	NA	NA	NA
Not confident/not confident at all	391	49.4	(44.3–54.4)	363	37.2	(32.2–42.4)	−12.2	NA	NA	0.97	(0.61–1.55)	0.90
Presence of symptoms of burnout												
No	274	45.6	(39.6–51.7)	250	33.2	(27.5–39.5)	−12.4	NA	NA	NA	NA	NA
Yes	180	53.3	(45.8–60.7)	168	44.0	(36.5–51.9)	−9.3	NA	NA	1.18	(0.84–1.67)	0.34
Vaccine hesitancy, mean (SD)												
Complacency	454	3.20 (1.19)		418	3.20 (1.27)		NA	NA	NA	0.72	(0.62–0.85)	0.00
Constraint	454	2.92 (1.28)		418	2.97 (1.25)		NA	NA	NA	0.90	(0.78–1.05)	0.18
Calculation	454	5.64 (0.96)		418	5.78 (0.88)		NA	NA	NA	0.87	(0.72–1.05)	0.15
Confidence	454	4.60 (1.19)		418	4.42 (1.30)		NA	NA	NA	1.71	(1.48–1.99)	0.00
Collective	454	5.46 (1.16)		418	5.52 (1.16)		NA	NA	NA	1.31	(1.10–1.55)	0.00

*aOR, adjusted odds ratio; NA, not applicable; SD, standard deviation.
†Based on the partially overlapping samples *z*-test; valid only for non–time-varying participant characteristics.

Appendix Table 9. Comparison of diseases in terms of perceived severity, Hong Kong, 2020

Diseases	Round 1 (N = 2,478)		Round 2 (N = 644)		Round 3 (N = 542)		Round 4 (N = 484)		Round 5 (N = 441)		p (trend)
	n	%	n	%	n	%	n	%	n	%	
Emerging infectious diseases											
COVID-19											0.00
Very bad/bad	2,061	98.8	620	99.5	523	98.9	442	95.5	407	95.8	
Neutral/not bad/not bad at all	26	1.2	3	0.5	6	1.1	21	4.5	18	4.2	
Existing infectious diseases											
Seasonal influenza											0.00
Very bad/bad	1,389	66.6	377	60.5	334	63.1	252	54.4	255	60.0	
Neutral/not bad/not bad at all	698	33.4	246	39.5	195	36.9	211	45.6	170	40.0	
2009 influenza pandemic											0.00
Very bad/bad	1,830	87.7	525	84.3	444	83.9	367	79.3	333	78.4	
Neutral/not bad/not bad at all	257	12.3	98	15.7	85	16.1	96	20.7	92	21.6	
2003 SARS											0.28
Very bad/bad	2,051	98.3	618	99.2	518	97.9	454	98.1	414	97.4	
Neutral/not bad/not bad at all	36	1.7	5	0.8	11	2.1	9	1.9	11	2.6	
Noncommunicable diseases											
Diabetes											0.00
Very bad/bad	1,772	84.9	576	92.5	494	93.4	422	91.1	397	93.4	
Neutral/not bad/not bad at all	315	15.1	47	7.5	35	6.6	41	8.9	28	6.6	
Cancer											0.00
Very bad/bad	2,002	95.9	612	98.2	518	97.9	455	98.3	420	98.8	
Neutral/not bad/not bad at all	85	4.1	11	1.8	11	2.1	8	1.7	5	1.2	
Heart disease											0.00
Very bad/bad	1,966	94.2	606	97.3	519	98.1	453	97.8	414	97.4	
Neutral/not bad/not bad at all	121	5.8	17	2.7	10	1.9	10	2.2	11	2.6	
AIDS											0.01
Very bad/bad	1,957	93.8	602	96.6	516	97.5	447	96.5	406	95.5	
Neutral/not bad/not bad at all	130	6.2	21	3.4	13	2.5	16	3.5	19	4.5	

Appendix Table 10. Confidence in the local government and doctors, Hong Kong, 2020

Area	Round 1 (N = 2,478) n	Round 1 %	Round 2 (N = 644) n	Round 2 %	Round 3 (N = 542) n	Round 3 %	Round 4 (N = 484) n	Round 4 %	Round 5 (N = 441) n	Round 5 %	p (trend)
Confidence in the local government											
COVID-19 information provided											0.00
Very confident/confident	148	6.5	59	9.4	72	13.5	60	12.8	48	11.2	
Neutral/not confident/not confident at all	2,117	93.5	571	90.6	462	86.5	409	87.2	380	88.8	
Measures taken in response to COVID-19											0.07
Very confident/confident	58	2.6	17	2.7	33	6.2	16	3.4	11	2.6	
Neutral/not confident/not confident at all	2,207	97.4	613	97.3	501	93.8	453	96.6	417	97.4	
Ability to deal with COVID-19											0.59
Very confident/confident	74	3.3	20	3.2	36	6.7	13	2.8	13	3.0	
Neutral/not confident/not confident at all	2,191	96.7	610	96.8	498	93.3	456	97.2	415	97.0	
Decisiveness in dealing with COVID-19											0.16
Very confident/confident	31	1.4	10	1.6	21	3.9	7	1.5	7	1.6	
Neutral/not confident/not confident at all	2,234	98.6	620	98.4	513	96.1	462	98.5	421	98.4	
Itself, apart from ability to manage major crises											0.45
Very confident/confident	47	2.1	15	2.4	16	3.0	9	1.9	11	2.6	
Neutral/not confident/not confident at all	2,218	97.9	615	97.6	518	97.0	460	98.1	417	97.4	
Confidence in doctors											
Ability to diagnose COVID-19											0.00
Very confident/confident	1,171	53.3	349	55.6	352	66.3	302	64.7	298	69.6	
Neutral/not confident/not confident at all	1,025	46.7	279	44.4	179	33.7	165	35.3	130	30.4	

Appendix Table 11. Knowledge of route of transmission of COVID-19, Hong Kong, 2020

Knowledge level	Round 1 (N = 2,478) n	Round 1 %	Round 2 (N = 644) n	Round 2 %	Round 3 (N = 542) n	Round 3 %	Round 4 (N = 484) n	Round 4 %	Round 5 (N = 441) n	Round 5 %	p (trend)
Keep an eye on COVID-19 progress											
Yes	2,309	99.4	625	98.7	517	96.6	463	98.5	419	97.4	0.00
No	15	0.6	8	1.3	18	3.4	7	1.5	11	2.6	
Actively search for COVID-19 information											0.00
Yes	1,933	83.2	500	79.0	343	64.1	353	75.1	301	70.0	
No	391	16.8	133	21.0	192	35.9	117	24.9	129	30.0	
Understanding of COVID-19											0.00
Very well/well	763	35.2	35	5.6	36	6.8	24	5.1	22	5.1	
Neutral/Not well/Not well at all	1,403	64.8	591	94.4	493	93.2	443	94.9	406	94.9	
COVID-19 Infection likelihood for the following routes											
Talk to an asymptomatic infected person without physical touch											0.91
Very likely/likely	2,010	92.8	596	95.2	496	93.8	440	94.2	393	91.8	
Neutral/unlikely/very unlikely	156	7.2	30	4.8	33	6.2	27	5.8	35	8.2	

Knowledge level	Round 1 (N = 2,478) n	%	Round 2 (N = 644) n	%	Round 3 (N = 542) n	%	Round 4 (N = 484) n	%	Round 5 (N = 441) n	%	p (trend)
Talk to a symptomatic infected person without physical contact											0.70
Very likely/likely	2,085	96.3	613	97.9	506	95.7	455	97.4	412	96.3	
Neutral/unlikely/very unlikely	81	3.7	13	2.1	23	4.3	12	2.6	16	3.7	
Physical touch with infected but asymptomatic persons											0.14
Very likely/likely	2,057	95.0	611	97.6	511	96.6	453	97.0	410	95.8	
Neutral/unlikely/very unlikely	109	5.0	15	2.4	18	3.4	14	3.0	18	4.2	
Physical touch with infected and symptomatic persons											0.03
Very likely/likely	2,084	96.2	615	98.2	519	98.1	458	98.1	418	97.7	
Neutral/unlikely/very unlikely	82	3.8	11	1.8	10	1.9	9	1.9	10	2.3	
Droplets											0.45
Very likely/likely	2,134	98.5	620	99.0	517	97.7	460	98.5	420	98.1	
Neutral/unlikely/very unlikely	32	1.5	6	1.0	12	2.3	7	1.5	8	1.9	
Aerosol when infected persons cough or sneeze											0.01
Very likely/likely	1,920	88.6	556	88.8	483	91.3	440	94.2	389	90.9	
Neutral/unlikely/very unlikely	246	11.4	70	11.2	46	8.7	27	5.8	39	9.1	
Virus-contaminated environment											0.86
Very likely/likely	2,064	95.3	598	95.5	499	94.3	445	95.3	410	95.8	
Neutral/unlikely/very unlikely	102	4.7	28	4.5	30	5.7	22	4.7	18	4.2	
Eating game (wild animals)											0.00
Very likely/likely	2,006	92.6	540	86.3	468	88.5	363	77.7	314	73.4	
Neutral/unlikely/very unlikely	160	7.4	86	13.7	61	11.5	104	22.3	114	26.6	
Wet market											0.00
Very likely/likely	1,750	80.8	422	67.4	366	69.2	356	76.2	311	72.7	
Neutral/unlikely/very unlikely	416	19.2	204	32.6	163	30.8	111	23.8	117	27.3	
Eating seafood imported from Wuhan											0.00
Very likely/likely	1,569	72.4	381	60.9	305	57.7	274	58.7	236	55.1	
Neutral/unlikely/very unlikely	597	27.6	245	39.1	224	42.3	193	41.3	192	44.9	
Eating/using other products imported from Wuhan											0.00
Very likely/likely	1,441	66.5	352	56.2	265	50.1	264	56.5	219	51.2	
Neutral/unlikely/very unlikely	725	33.5	274	43.8	264	49.9	203	43.5	209	48.8	
